# Interfamily Grafted Hybrids *Vitis vinifera*/*Schisandra chinensis* Resulted in Transcriptomic, Phenotypic, and Metabolic Changes

**DOI:** 10.3390/plants13121676

**Published:** 2024-06-17

**Authors:** Shulin Zhang, Zhuo Chen, Junhui Zhao, Songfeng Diao, Li Tian, Ying Zhao, Fangdong Li, Gao-Pu Zhu

**Affiliations:** 1 School of Horticulture and Landscape Architecture, Henan Institute of Science and Technology, Xinxiang 453003, Chinachen23330223@163.com (Z.C.);; 2 Research Institute of Non-Timber Forestry, Chinese Academy of Forestry, Zhengzhou 450003, Chinalifangdong66@163.com (F.L.); 3 College of Biology and Food Engineering, Anyang Institute of Technology, Anyang 455000, China; litian@ayit.edu.cn; 4 CAS Key Laboratory of Tropical Plant Resources and Sustainable Use, Xishuangbanna Tropical Botanical Garden, The Innovative Academy of Seed Design, Chinese Academy of Sciences, Kunming 650223, China; 5 Guangxi Subtropical Crops Research Institute, Nanning 530001, China; zhaoying-222@163.com

**Keywords:** graft hybridization, distant-grafting, *Vitis vinifera*, transcriptome, miRNA, metabolome

## Abstract

Long-distance transfer of genetic material and metabolites between rootstock and scions is well documented in homo-grafted hybrids but has rarely been reported in genetically-distant grafts where the rootstock and scion belong to different families. In this study, we grafted *Vitis vinifera* scions onto *Schisandra chinensis* stocks and obtained 20 vegetative hybrids, *Vitis vinifera*/*Schisandra chinensis* (Vs). After 25 years of growth, we found that the phenotypes of the leaves, internodes, and fruits of the Vs hybrids above the graft union resembled an intermediate phenotype between *V. vinifera* and *S. chinensis,* and the new traits were stable when propagated vegetatively. We further analyzed genetic differences between Vv plants and Vs hybrids using high-throughput sequencing, while metabolomes were analyzed by liquid chromatography-mass spectrometry (LC-MS). We found a total of 2113 differentially expressed genes (DEGs). GO annotation and KEGG pathway enrichment analysis showed that these DEGs enriched mainly in oxidation-reduction and metabolic processes. Seventy-nine differentially expressed miRNAs (DEMs) containing 27 known miRNAs and 52 novel miRNAs were identified. A degradation analysis detected 840 target genes corresponding to 252 miRNAs, of which 12 DEMs and their corresponding target gene expression levels were mostly negatively correlated. Furthermore, 1188 differential metabolic compounds were identified. In particular, in Vs hybrids, the abundance of the metabolites schizandrin and gomisin as the main medicinal ingredients in *S. chinensis* were down-regulated and up-regulated, respectively. Our data demonstrated the effects of interfamily grafts on the phenotype, transcript profile and metabolites of the scion, and also provided new insight into the genetic, phenotypic, and metabolic plasticity associated with genetically distant grafted hybrids.

## 1. Introduction

Grafting is a commonly used method in forestry reproduction and is an important technique to bestow beneficial traits in fruit tree cultivation [1]. Graft hybridization is a type of asexual hybridization in which grafting can induce heritable changes and serves as a new method for germplasm innovation [2,3,4]. With the development of molecular biology and genetic resources for genetic stocks of interest, it has been shown that long-distance transport of mRNA, small RNAs, proteins, and hormones can occur at graft junctions [5,6,7,8]. A study on Chinese white pear, *Pyrus bretschneideri,* grafted on wild pear, demonstrated that mRNAs can be transported bidirectionally of the grafting rootstock and scion [9]. Small RNAs can be transmitted and coordinate plant signal transmission through the connection of the phloem tissues at the grafting site [3,10,11]. A recent study of wild cabbage, *Brassica oleracea*, grafted onto brown mustard, revealed that the graft altered the methylation levels of some phenotype-related genes in the offspring [3,10,11]. The FT protein could induce plant flowering via long-distance movement in the phloem at the graft junction [12]. In tomato, *Solanum lycopersicum*, the rootstock-derived ethylene precursor ACC was transferred to the scion via the graft junction and was associated with increased water use efficiency of the tomato chimeras and improved growth in soil lacking potassium [13,14,15].

Grafting has mostly been used between plants of the same species and different species within the same genus. Some combinations of different genera in the same family, such as *Armeniaca vulgaris* + *Amygdalus persica*, *Armeniaca vulgaris* + *Cerasus japonica* [2], and *Lycium barbarum* + *Lycopersicon esculentum* [16], are also relatively easy to produce. However, most examples of interfamily grafted hybrids of woody plants only come from historical documents, and there are no living plants or research materials that serve as direct evidence of such hybrids.

Grafting is extremely important in grape cultivation and has a long propagation history. In China, the book “Agricultural Must Use” written by Yi Wu during the early Southern Song Dynasty (in 1127–1279) recorded a method for grafting *V. vinifera* and jujuba (*Ziziphus jujuba*), which improved fruit quality. Until the late 1800s, insects in the genus *Phylloxera* caused substantial losses to the French wine industry until a resistant rootstock was found [17]. Since then, researchers all over the world have achieved significant progress in improving the environmental adaptability, growth vigor, and fruit quality of the scion by grafting to different grape variety rootstocks [7,18,19]. We repeatedly grafted *V. vinifera* scions onto *S. chinensis* stocks and obtained vegetative hybrids (*Vitis vinifera*/*Schisandra chinensis*) that were able to grow normally for over 25 years. The *Vitis* + *Schisandra* fruit grafting hybrid demonstrated the feasibility of grafting woody plants belonging to different families or higher taxa, which could produce edible grapes with the medicinal properties of *S. chinensis*. Furthermore, we compared the differences between interfamily hybrid *Vitis vinifera*/*Schisandra chinensis* (Vs) and self-rooted grapes (Vv) in terms of mRNA, miRNA, and the degradome using high-throughput sequencing and characterized the metabolomes using liquid chromatography-mass spectrometry (LC-MS). This study provides a reference for future research on berry trees by distant-grafting hybridization in berry trees.

## 2. Materials and Methods

### 2.1. Study Site, Plant Materials and Phenotype Analysis

This study was conducted in Si Mountain National Forest Park, Xixia County, Henan Province (Geographical location: 33°17′31″ N, 111°28′16″ E). The soil type at the study site was red clay with Ph > 7, and the main climate type in the region was a warm continental monsoon climate. The average duration of sunshine and the average temperature are 2019 h and 15.0 °C, respectively. The non-frost period was 236.2 d.

Scions from *V. vinifera* ‘Hongruibao’ were grafted onto wild *S. chinensis* rootstocks to construct *V. vinifera*/*S. chinensis* graft hybrids (Vs) using split grafting method. Meanwhile, self-rooted grafting from *V. vinifera* ‘Hongruibao’ (Vv) and wild *S. chinensis* (Sc) were also constructed as controls. Finally, a total of 23 viable vegetative hybrids were obtained, and anthesis and frutescence times of Vs were on 20 April and 28 June, while anthesis and frutescence times of Vv were on 28 April and 15 July, and anthesis and frutescence times of *Sc* were on 10 April and 25 July, respectively. However, the fruiting period comes after grafting 2–3 years.

Twenty-five years after grafting, the phloem without xylem was isolated using a scalpel, and samples of fruits and functional mature leaves in the fully ripe stage were packed in silver paper and stored in liquid nitrogen until use. Grafting sites were sampled with FAA stationary liquid and paraffin sections. The differences in internode length and the length, width, and weight of fruits were measured using Vernier calipers (0.01 mm) or an electronic scale (0.01 g). Data were analyzed using Student *t*-tests to compare the two groups with the software DPS 7.05 [20]. The significance level was set at *p* ≤ 0.01.

### 2.2. Transcriptome Sequencing and Analyses

Transcriptomic libraries were constructed from total mixed RNA pools (concentration: 40 ng/μL) consisting of RNA extracted from leaves, phloem, and whole ripe berries from Vs hybrids and Vv scions. Every group included samples from three randomly selected hybrid plants (three biological replicates). Total RNA and miRNA were extracted using a Polysaccharide Polyphenol Plan MicroRNA Extraction Kit following the manufacturer’s protocol. RNA quantity and purity were determined using 1% agarose gel electrophoresis and a Bioanalyzer 2100 (Agilent Technologies, Waldbronn, Germany). RNA with a concentration of 200 µg/mL or greater, a 28S rRNAs/18S rRNAs ratio over 2, and an RNA Integrity Number over 7 were defined as suitable samples for future experiments. A cDNA library constructed using pooled RNA was sequenced with the Illumina Hiseq4000 sequence platform. We sequenced the transcriptome using the Illumina paired-end RNA-seq approach. This yielded 43.68 GB of transcriptomic sequences. Prior to assembly, the low-quality reads (reads containing sequencing adaptors, reads containing sequencing primers, and nucleotides with a q-quality score lower than 20) were removed. Next, a total of 43.3 GB of cleaned, paired-end reads were produced. We aligned reads to the *V. vinifera* genome using the HISAT package [21]. The mapped reads of each sample were assembled using StringTie (version 1.3.4) [22]. Then, all transcriptomes were merged to reconstruct a comprehensive transcriptome using ptcerl scripts. After the final transcriptome was generated, StringTie was used to estimate the expression levels of all transcripts. StringTie was used to compute expression levels for mRNAs by calculating FPKM (Fragments per Kilobase Million). The significantly differentially expressed genes (DEGs) between the two samples were judged by the false discovery rate of <0.05, *p*-value < 0.005, and |log2FC| > 1. All unigenes were subjected to an NCBI search (GSE 130209). GO (Gene Ontology) (http://geneontology.org, accessed on 23 July 2020) and KEGG (Kyoto Encyclopedia of Genes and Genomes annotations) (http://www.kegg.jp/kegg, accessed on 10 August 2020) [23] enrichment searches were conducted using the BLASTX algorithm. These significantly enriched functional clusters were determined by a corrected *p*-value of < 0.05. The figures showing significant enrichment of GO and KEGG classifications were constructed using the ggplot2 package in the R package-Ballgown [24].

### 2.3. Small RNA Sequencing, miRNA Identification, and Functional Prediction

Small RNA sequencing libraries of 300 ng/μL were established using TruSeq^TM^ Small RNA Sample Prep Kits (Illumina, San Diego, CA, USA). The constructed library was sequenced using the Illumina Hiseq 2000/2500. The raw reads were analyzed by ACGT101-miR (LC Sciences, Houston, TX, USA) as follows: 3′-joints and garbage sequences were removed, and sequences with a base length of 18–25 nt were retained. The remaining sequences were compared to mRNA (without miRNA), RFam, and Repbase databases to filter other RNAs. Next, effective data were identified by comparison with precursor genomes to identify miRNAs. Finally, differential miRNA analysis was performed, and differential miRNA target gene prediction analysis was conducted. The differentially-expressed miRNA (DEMs) between the two groups were assessed by the *p*-value (*p* < 0.1, *p* < 0.05, *p* < 0.01). The predicted target genes of the differential miRNAs were then annotated within the GO and KEGG databases.

### 2.4. Degradome Sequencing and Target Identification

Equal parts of the six samples of leaves, fruits, and phloem tissue RNAs were mixed, respectively (final concentration: 200 ng/μL) and sent to Hangzhou LC-bio -Co., Ltd. (Hangzhou, China) for cDNA library construction and Illumina Hiseq2000/2500 sequencing. Target gene prediction was performed using the CleaveLand Version 3.0 program, and Oligo map short reading frame calibrator to detect mRNAs that matched the sequences of the degradation group [25,26]. Then, the degradome reads were mapped to the transcriptome data. Target gene prediction was performed using the Cleave-Land program, and Oligo map short reading frame calibrator to find mRNAs that matched the sequences of the degradation group [27]. 

The targets were categorized as 0, 1, 2, 3, and 4, as in a previous study, based on the following criteria [28]. Category 0 refers to when there was more than one raw read at a position, which indicates that abundance at the position is equal to the maximum on the transcript. Category 1 refers to when there is more than one maximum on the transcript. Category 2 refers to when there is more than one raw read at a position, which indicates that abundance at the position is less than the maximum but higher than the median for the transcript. Category 3 refers to when there is more than one raw read at a position, which indicates that abundance at the position is equal to or less than the median for the transcript. Category 4 indicates that there is only one raw read at the position. The potential miRNA targets were built by psRNATarget, and we predicted and analyzed the results of the degradation group sequencing and created a t-plot of the degraded group. GO and KEGG enrichments were used to obtain the potential function and classification of targets.

### 2.5. Quantitative Real-Time RT-PCR (RT-qPCR) Validation

To examine the sequencing results and reveal the differential expression of genes in different tissues, we selected 11 mRNAs each from leaves, fruits, and phloem and 10 miRNAs from mixed samples of the two groups for RT-qPCR analysis. The RNA extraction and quality detection methods were the same as described above. Reverse transcription and RT-qPCR for mRNAs were conducted using the EasyScript One-Step gDNA Removal and cDNA Synthesis SuperMix kit (and TransStart Top Green qPCR SuperMix kits from Transgene Biotech Co., Ltd., Beijing, China). The primers were designed using Primer3 web version 4.1.0 (http://primer3.ut.ee/ accessed on 10 August 2020). EF1α was selected as an internal reference gene [29]. The miRNA reverse transcription, and RT-qPCR were conducted using a miRNA cDNA Synthesis Kit, with Poly(A) Polymerase Tailing and BrightGreen miRNA qPCR MasterMix-No Dye (Applied Biological Materials, Inc., Vancouver, BC, Canada). The specific miRNA forward primers were designed according to the sequence of the respective miRNAs, and the reverse primer used was Universal 3′ miRNA Reverse Primer (Applied Biological Materials Inc., Vancouver, BC, Canada). We used 5.8S as the internal reference gene for miRNA [30]. The gene names, sequences, and primers used for RT-qPCR are listed in Table S1. The RT-qPCR reactions were performed in 96-well clear Multiplate^TM^ PCR Plates (Bio-Rad, Hercules, CA, USA) in a Bio-Red CFX96 machine. Three repetitions were conducted for each RT-qPCR reaction. The experimental results were analyzed using the 2^−△△CT^ method [31]. qPCR was performed on leaves, berries, and phloem RNA of Vv and Vs, respectively, and the results were generally consistent with the sequencing results.

### 2.6. Metabolomic Analysis

First, five samples of ripe fruit (without seeds) were respectively placed in 1.5 mL cryogenic vials and thawed in a liquid nitrogen box before extraction. Then, every 100 mg of each fruit sample was weighed and ground in liquid nitrogen. Next, samples were mixed and brought up to 120 mL in 50% methanol and incubated at 25 °C for 10 min. Subsequently, the extraction mixture was then stored overnight at −20 °C to allow proteins to precipitate. After centrifugation at 4000× *g* for 20 min, the supernatants were transferred into new 96-well plates. The samples were stored at −80 °C before LC-MS analysis. All samples were processed by the LC-MS system following manufacturer recommendations, and the loading quantity of each sample was 10 mL. The samples were scanned with negative and positive mass spectrometry. To evaluate the stability of the LC-MS during the entire acquisition, a quality control (QC) sample (pool of all samples) was acquired after every 10 samples. The acquired MS data pretreatments, including peak picking, peak grouping, retention time correction, second peak grouping, and annotation of isotopes and adducts, were performed using the XCMS software(online). The LC−MS raw data files were converted into mzXML format and then processed by the XCMS, CAMERA, and metaX toolbox using R software. Each ion was identified by combining retention times (RT) and *m*/*z* data. The intensity of each peak was recorded, and the three-dimensional matrix containing arbitrarily assigned peak indices (retention time-*m*/*z* pairs), sample names (observations), and ion intensity information (variables) was generated. To screen for differential metabolites, univariate analysis of variance multiple (fold-change) and *t*-tests were used to obtain a q-value by BH correction, combined with the multivariate statistical analysis of the VIP value of PLS-DA. Different ions simultaneously satisfied the following criteria: ratio > 2 or ratio < 1/2; q-value < 0.05; VIP > 1. The online KEGG database was used to annotate the metabolites by matching the exact molecular mass data (*m*/*z*) of samples with those from the database. Wilcoxon tests were conducted to detect differences in metabolite concentrations among the three groups. The *p*-value was adjusted for multiple tests using a false discovery rate procedure. The variable important for the projection (VIP) value was calculated. A VIP cut-off value of 1.0 was used to select important features.

## 3. Results

### 3.1. Phenotype Analysis of Vitis vinifera/Schisandra chinensis Hybrid

**Figure 1:**
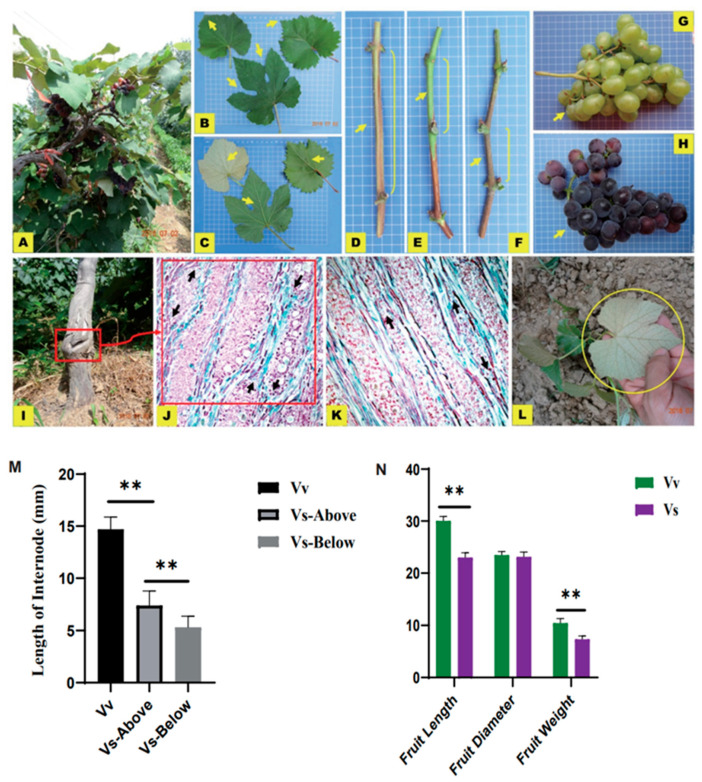
The phenotype of vegetative: *Vitis vinifera*/*Schisandra chinensis* hybrids (**A**) The vegetative hybrids of *Vitis vinifera*/*Schisandra chinensis*; (**B**) The upper-side of a leaf of vegetative hybrids above the graft union (top left), self-rooted *V. vinifera* ‘Hongruibao’ (top right), and vegetative hybrids below the graft union (centre down); (**C**) The lower-side of leaves correspond with figure (**B**); (**D**) Internode of self-rooted *V. vinifera* ‘Hongruibao’; (**E**) Internode of vegetative hybrids above the graft union; (**F**) Internode of vegetative hybrids below the graft union; (**G**) Fruits of self-rooted *V. vinifera* ‘Hongruibao’; (**H**) Fruits of vegetative hybrids of *Vitis vinifera*/*Schisandra chinensis*; (**I**) Graft junction; (**J**) Microstructure of graft junction in vegetative hybrids of *Vitis vinifera*/*Schisandra chinensis*, showing the bridges of vascular bundles; (**K**) Microstructure of self-rooted *V. vinifera* ‘Hongruibao’, showing the vascular bundle; (**L**) The cuttage seedling of vegetative hybrids of *Vitis vinifera*/*Schisandra chinensis*, showing stable genetics of vegetative hybrids traits. The arrows show the significant differences between scion and stock, and the scale plate of background for Figure (**B**–**H**) indicate 1 cm; (**M**,**N**) Statistical analysis of Vv and Vs internode length and fruits. The ordinate quantity of N is in millimeters (Fruit Length and Fruit Diameter) or grams (Fruit Weight). The asterisks showed highly significant differences according to Student’s *t*−test (*p* < 0.01). Error bars indicate SD (*n* = 10).

The 20 vegetative hybrids of *Vitis vinifera*/*Schisandra chinensis*(VS) were grown normally for 25 years (Figure 1A). The leaves of Vs hybrids above the graft junction were significantly different from those of self-rooted *V. vinifera* ‘Hongruibao’ (Figure 1B,C): the leaf apex of the hybrids was more obtuse, and the abaxial side was covered with shorter and whiter hairs (Figure 1B,C), while below the graft union, the Vs hybrids produced five dehiscent leaves, and the veins were radial rather than reticulate with highly-visible veins (Figure 1B,C). The most notable characteristic of Vs hybrids was on the abaxial side of the leaves above the graft union, which were white (Figure 1C, top left), while the leaves of Vv plants and Vs hybrids below the graft union were dark green (Figure 1C).

The internode of self-rooted *V. vinifera* ‘Hongruibao’ was the longest (Figure 1D) at 14.7 cm and had a high trichome density. Meanwhile, the internode of Vs hybrids above the graft union was of medium length at 7.4 and displayed a smooth surface (Figure 1E). and Vs hybrid internodes below the graft union were the shortest at approximately 5.3 cm (Figure 1F), all of which were significantly different (Figure 1M). Interestingly, the internode length of vegetative hybrids was intermediate between rootstocks and scions.

The fruits of self-rooted *V. vinifera* ‘Hongruibao’ were oval and a lighter green color (Figure 1G), while the fruits of Vs hybrids were round and more purple or red than those of *V. vinifera* ‘Hongruibao’ (Figure 1H) with a sourer taste. Both types of fruits were covered with thick wax, but Vs hybrid cuticles appeared thicker than the self-rooted Vv. The difference in terms of the length (30.07 ± 1.14 mm) and weight (10.46 ± 1.15 g) of mature fruits of Vv plants differed significantly (*p* ≤ 0.01) from those of Vs hybrids (23.06 ± 1.21 mm and 6.86 ± 0.82 g, respectively), but there were no significant differences in the widths of the fruits (Figure 1N).

The graft unions were swollen (Figure 1I), and the anatomical structure indicated the formation of numerous bridges of the vascular bundle (Figure 1J), but those of the self-rooted *V. vinifera* ‘Hongruibao’ were not obvious (Figure 1K). At the 200 μm scale, the numerous vascular bundle bridges from hybrids Vs were more extensive than in self-rooted Vv (Figure 1I–K). In total, the area above and below the graft union was similar, indicating that the rootstock and the scions were concordant and compatible.

We reproduced the Vs hybrids using twig cuttings above the graft junction in a garden in June 2018 and replicated the traits of the seedlings, including leaf type, color, and hairs on the reverse side of leaves. The radial veins were stable in the adult tree (Figure 1L), which indicates stable inheritance. However, the rate of survival in these Vs hybrids was 15–20%, which was much lower than that of *V. vinifera* ‘Hongruibao’ (80–85%), suggesting there are some barriers in grafting these hybrids.

### 3.2. Transcriptome Sequencing and Functional Annotation of DEGs

Since there were many morphological differences between heterografted Vs and self-grafted Vv grapes, we hypothesized that heterografting may result in significant changes in genetic information and performed transcriptome sequencing on tissues from the scions of Vv and Vs. A total of 43.68 GB of sequencing data contained 291,145,166 raw reads. Clean data were obtained by filtering unqualified sequences using Cutadapt [32], after which 288,679,444 reads remained. Hisat [22] was used for the reference genome comparison of clean data and produced 30,609 genes from 131,224,950 unique mapped reads. The summary of the Illumina Hiseq4000 transcriptome sequencing of the two groups is shown in Table 1. The screening criteria for DEGs were |log2FC| > 1 and *p* < 0.005. There were 222 DEGs; the up– and down–regulated DEGs are shown in Table S2. The GO annotation grouping was arranged from high to low according to the number of annotated target genes and is presented in Table S2. The five most abundant transcripts involved with biological processes were associated with oxidation-reduction, metabolic processes, protein phosphorylation, regulation of transcription, and transport. Among the 15 cellar components, the three most highly-represented were the membrane, integral components of the membrane, and the nucleus. Of the 10 molecular function categories, the two most significant categories were ATP binding and oxidoreductase activity.

To better understand the biological roles of these genes, we submitted them for KEGG annotation (Table S2). KEGG enrichment analysis showed that the degradome-enriched pathways were mainly associated with carbon metabolism, ribosomes, biosynthesis of amino acids, protein processing in the endoplasmic reticulum, glyoxylate and dicarboxylate metabolism, photosynthesis, and glycolysis and gluconeogenesis.

**Figure 2:**
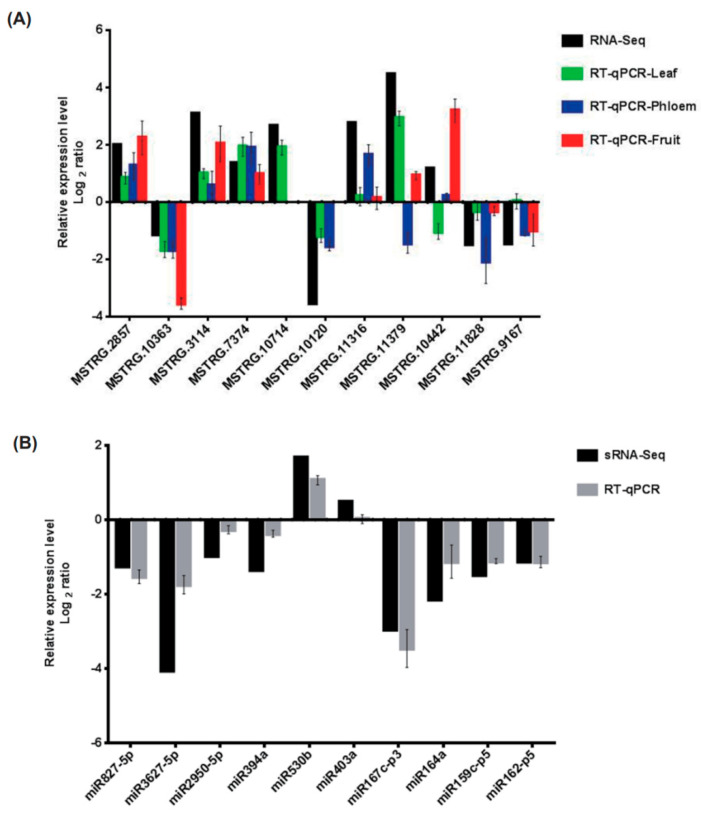
Validation of DEGs and DEMs expression level. (**A**) The relative expression levels of 11 DEGs were verified in different tissues by transcriptome and qPCR analysis; (**B**) the relative expression levels of 10 DEMs were verified by transcriptome and qPCR analysis. Up−regulation and down-regulation indicate the expression level of *Vitis vinifera*/*Schisandra chinensis* (Vs) relative to *Vitis vinifera* (Vv). Three technical iterations and three biological replicates were conducted. The fifth DEG was too low in phloem and fruit, and the sixth DEG was too low in fruit; thus, their qPCR results are not shown.

We collected leaves, fruits, and phloem of the scions for RT-qPCR experiments to verify the sequencing results. The RT-qPCR results were consistent with our sequencing results. While some genes were up-regulated or down-regulated in all three parts, some genes were up-regulated or down-regulated in only one or two organs (Figure 2A).

### 3.3. Small RNA-Seq and Identification of miRNAs

**Figure 3:**
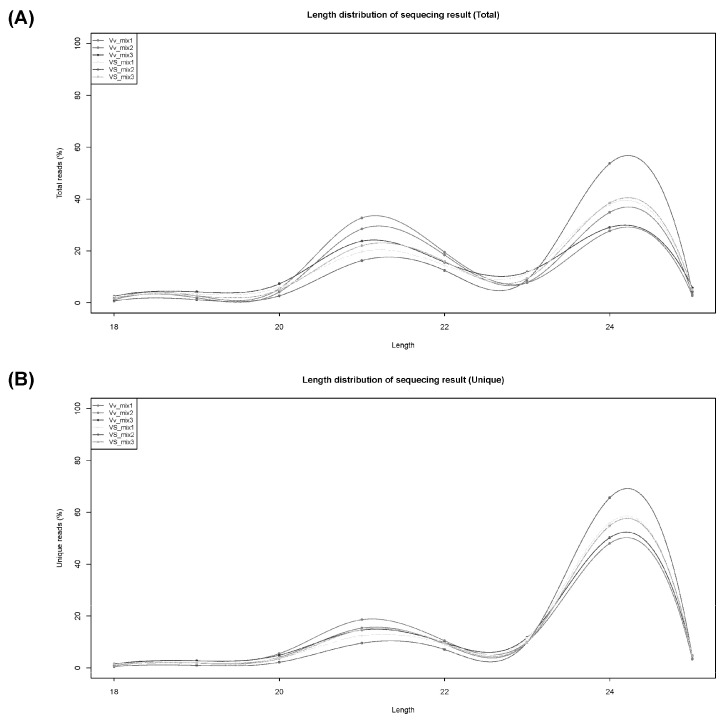
Length distribution of sRNA. (**A**) Length of distribution of total reads; (**B**) Length of distribution of unique reads. The abscissa represents the number of nucleotides, and the ordinate represents the fraction of sRNA.

Many genes are regulated by miRNAs, so we conducted miRNA sequencing to search for miRNAs that might play key functions in heterologous grafting. The small RNA library was assembled from mixed miRNA pools following the same procedures used for transcriptome sequencing. Identification of *V. vinifera* miRNAs and their targets was performed using high-throughput sequencing and degradome analysis using previously reported methods 9 [33,34,35,36] and then compared to the Pfam database [37]. The length distribution of unique sRNAs (small RNAs) is summarized in Figure 3, and most sRNAs are 24 nt in length.

The unique sequences mapped to species-specific mature miRNAs in hairpin arms were identified as known miRNAs. The unique sequences mapping to the other arm of known precursor hairpins opposite to the annotated arm were considered to be novel 5p- or 3p-derived miRNA candidates. The remaining sequences were mapped to other selected species precursors (with the exclusion of specific species) in miRBase 21.0 using a BLAST search. The mapped pre-miRNAs were further BLASTed against specific genomes to determine their genomic locations. The sequences were mapped to known miRNAs. Unmapped sequences were BLASTed against the specific genomes, and RNAs containing hairpin structures were predicted from the flanking 120 nt sequences using RNAfold software (http://rna.tbi.univie.ac.at/cgi-bin/RNAfold.cgi, accessed on 26 August 2020). Those that met the criteria in secondary structure predictions were considered to be potentially novel miRNA candidates. In total, 1195 miRNAs were found in the RNA library and categorized into six groups based on their degree of matching in miRbase. Group 1a (reads mapped to miRNAs/pre-miRNAs of *V. vinifera* in miRbase, and the pre-miRNAs were further mapped to the genome and EST) and Group 4 (reads that were not mapped to pre-miRNAs of selected species in miRbase but were mapped to the genome and extended genome sequences from the genome that were predicted to form hairpins) displayed the highest redundancy. RT-qPCR was used to verify the sequencing results, and the expression trend of most miRNAs was consistent with the sequencing results (Figure 2B).

### 3.4. Differential Expression Analysis and Function Annotation of miRNAs

To identify the effects of miRNAs in vegetative hybrids, the differential expression of miRNAs in six libraries of two groups was compared using the read counts generated from the high-throughput sequencing data (Table S3). In total, 79 miRNAs (*p* ≤ 0.05) containing 27 known miRNAs and 52 new miRNAs showed differential expression patterns. Among these, 43 miRNAs were up-regulated in Vs hybrids as compared to Vv plants. Furthermore, there were 42 DEMs, including 22 up-regulated and 20 down-regulated miRNAs. When the screening condition was relaxed to *p* ≤ 0.1, there were 83 up-regulated miRNAs and 76 down-regulated miRNAs. All detected miRNAs belonged to 46 gene families. Most miRNAs of the same gene family exhibited the same expression pattern. For example, the six miRNAs in the *miR162* family (*ptc-MIR162b-p5*, *vvi-MIR162-p5*, *vvi-miR162*, *tcc-miR162_R+1*, *hpe-miR162a_R+2*, and *stu-miR162a-5p_R+3_1ss4GT*) were down-regulated in Vs hybrids compared to Vv plants, and the five members in the *miR530* family (*ptc-MIR530a-p3_2ss12CA21GA*, *tcc-miR530b_L+2R-1_1*, *tcc-miR530b_R+1_1ss9CT*, *tcc-miR530b_L+2R-1_2*, and *stu-MIR530-p5*) were up-regulated in Vs hybrids compared to Vv plants. We also found some variable expression patterns between miRNAs within the same family. For example, there are 20 miRNAs in the *miR339* family; nine of which had low expression levels, another nine (*vvi-MIR399a-p5*, *vvi-miR399a*, *vvi-miR399e*, *vvi-MIR399h-p5*, *vvi-MIR399d-p5_1ss20TC*, *vvi-miR399d_L+2R-2*, *vvi-MIR399i-p5*, *vvi-miR399i*, and *tcc-miR399a_1ss21GT*) were down-regulated in Vs hybrids compared to Vv plants, while the remaining two (*vvi-MIR399g-p5* and *vvi-miR399*) were up-regulated. The cluster analysis data are shown in Table S3.

psRNATarget has been used to predict the target genes with significantly different expressed miRNAs [38]. Results of differential miRNA target gene prediction showed the target gene information corresponding to miRNA and provided the GO and KEGG annotation information of the target genes. GO Enrichment showed that target genes were mainly classified as being associated with defense responses, biosynthetic processes, salicylic acid biosynthesis, signal transduction, and drug transmembrane transporter activity (data shown in Table S4).

### 3.5. Target Prediction and Category Statistics of miRNAs by Degradome Sequencing and Targets Functional Enrichment

Because miRNAs mainly perform biological functions by degrading target genes, we performed degradome sequencing. Through degradome sequencing, 22,945,508 raw reads containing 5,922,957 unique reads were obtained from the mixed degradome pools. There were 14,163,908 (61.73% of all reads) transcript-mapped reads containing 4,607,411 unique transcript-mapped reads. The number of input transcripts was 52,365, and 45,728 transcripts were covered. All potential cleaved transcripts were divided into five categories according to the signature abundance at each occupied transcript position [28]. All resulting reads (t-signature) were reverse complemented and aligned to the miRNA identified in our study. The targets were selected and categorized as 0, 1, 2, 3, or 4.

In total, 1442 targets of 221 known miRNAs and 117 targets of 32 novel miRNAs were identified from degradome sequencing (target sites were detected by both target gene prediction and experimental sequencing results). Table S5 shows the sequencing results of the target genes predicted by miRNA that were detected by degradation and were statistically analyzed. Novel miRNAs and their targets are also shown in Table S5. Most of the miRNAs were found to cleave two or more different transcripts, whileath-*miR5658_R-3_1ss13AT* (73 targets) and *PC-5p-284876_17* (31 targets) represented the highest amounts of transcripts cleaved by known miRNAs in this study. Only 38 known miRNAs and 11 novel miRNAs cleaved a single transcript target (Table S6). Furthermore, the predictions of psRNATarget combined with the mRNA generated in the density file of the degradation group allowed the number of miRNAs that interacted with a transcript to be calculated. This showed that most of the transcripts (606 of 840) were cleaved by a particular miRNA, and one transcript target could be cleaved by no more than 11 miRNAs (Table S6). Target distribution among five categories revealed that 372 targets (approximately 24% of all targets) of conserved miRNAs belonged to category 0; 21 targets (approximately 1.35%) of conserved miRNAs belonged to category 1; 675 targets (approximately 43%) of conserved miRNAs belonged to category 2; 22 targets of conserved miRNAs belonged to category 3 (approximately 1.65%); and 469 targets (about 30%) of conserved miRNAs belonged to category 4 (Tables S5 and S6).

**Figure 4:**
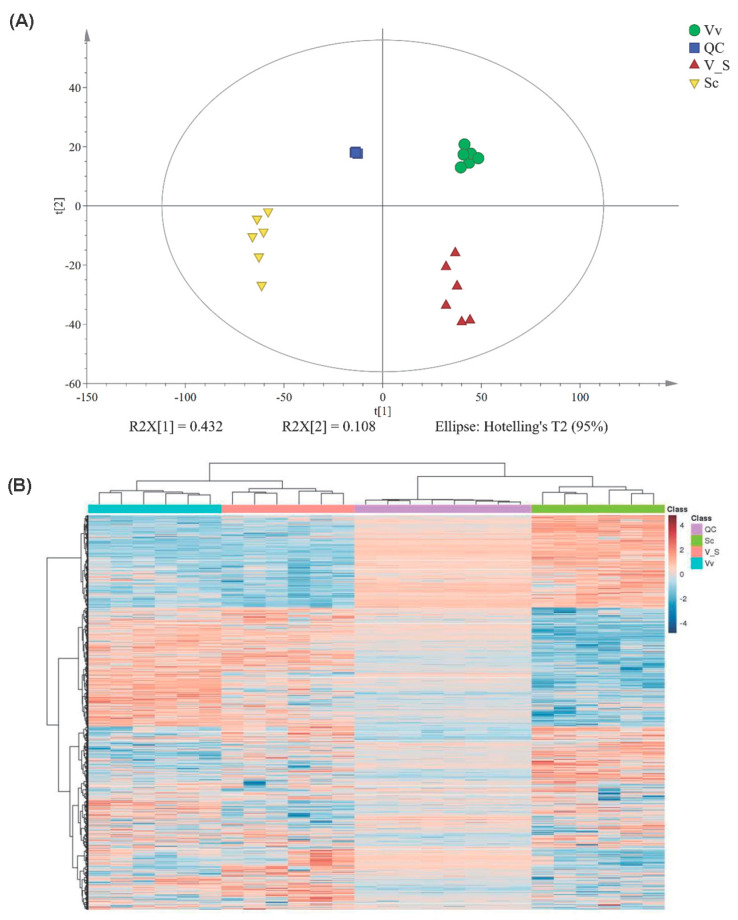
A complete analysis of several important pathway−related DEGs and DEMs affected by distant grafts. (**A**) Statistics on the up-regulation and down−regulation of genes associated with metabolism, oxidation-reduction processes, signal transduction, photosynthesis, and defense responses. Up− and down−regulation refers to the expression of *Vitis vinifera*/*Schisandra chinensis* (Vs) compared to that of *V. vinifera* (Vv). (**B**) Important DEGs and DEMs for related pathways. Biological pathways are listed in rectangles. Hexagons represent hormones (GA: gibberellin, IAA: auxin, BR: brassinosteroids). DEMs are represented by ellipses, DEGs are represented by tetrahedrons, and the transcript in parentheses is the ID of a gene preceding it. Because there are numerous DEGs, only the predecessors mentioned in the discussion and a few others with a *p*-value < 0.005 and FPKM > 5 in the sequence are listed. (The same color indicates the same class of substances or similar pathways, and neither DEGs nor DEMs are labeled as up-regulated or down-regulated, as it is unclear whether the function of each gene is promoted or inhibited).

Next, we analyzed target transcript homology to other species in our degradation library using the BLASTX algorithm. To further understand the functions of the identified target genes, we conducted a GO functional classification. Of the biological processes, the most abundant processes were the regulation of transcription, DNA templates, and auxin-activated signaling pathways. Among the cellar components, the three most highly represented were the nucleus, cytosol, and chloroplast stroma. Of the molecular function categories, the most highly represented categories were DNA binding, transcription factor activity, sequence-specific DNA binding, structural constituents of cell walls, and electron carrier activity. The up- and down-regulated genes and major DEGs and DEMs are depicted in Figure 4A, and the important DEGs and DEMs were mainly associated with defense responses, fruit development, signal transduction, photosynthesis, and metabolism pathways (Figure 4B).

### 3.6. Measurement of Metabolite Variation and Identification-Annotation 

**Figure 5:**
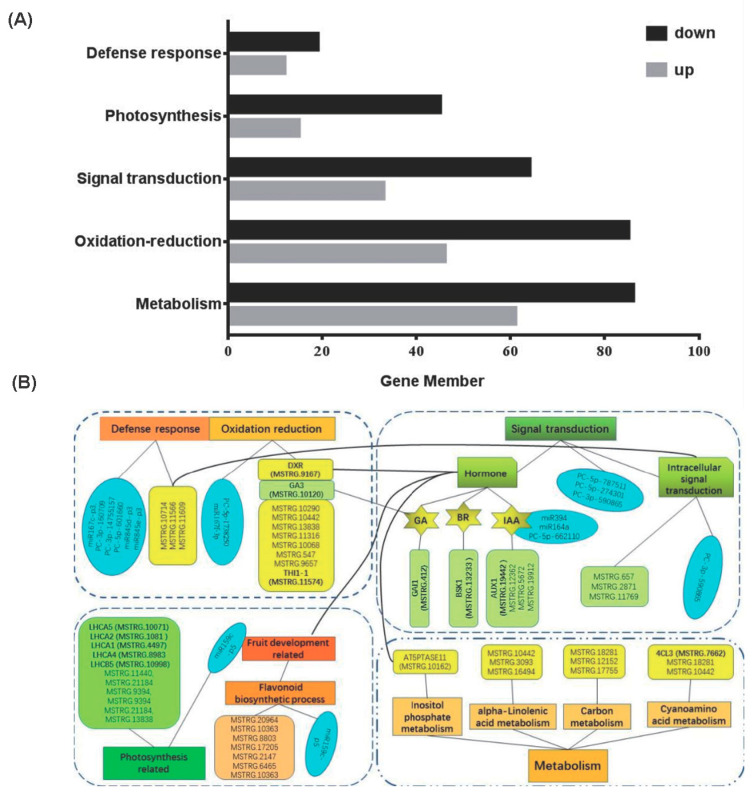
Metabolomic analysis of *Vitis vinifera*/*Schisandra chinensis* (Vs). (**A**) PCA score plot of metabolites in three types of samples and quality controls (QC). The red, green, blue, and violet represent QC, *Schisandra chinensis* (Sc), *Vitis vinifera*/*Schisandra chinensis* (Vs), and *Vitis vinifera* (Vv) samples, respectively. (**B**) Significant metabolites in different samples. The bold lines at the top indicate the type of sample: blue represents Vv, red represents Vs, purple represents QC, and green represents Sc. In the figure, blue and red represent down- and up-regulation.

Metabolically related DEGs and DEMs play a key role in plant grafting, and many small molecular metabolites can also be transported through the grafting site, thereby affecting the growth and development of organisms. In this study, we performed metabolomic sequencing analysis on mixed samples of fruit, phloem, and leaves, respectively, for Vv plants, Vs hybrids, and Sc plants via LC-MS (Table 2). Based on non-targeted metabolomics, principal component analysis (PCA) was performed to reveal the variation in the groups of metabolites. The results showed that the first two principal components accounted for 40.19% and 10.41% of the metabolic variance for all samples during negative ion mode mass spectrometry, and these values were 43.19% and 10.84% in positive ion mode, respectively (Figure 5A). According to the PCA score plot, the three groups were sharply divided in positive mass spectrometry, with Sc plants being the farthest. In negative mass spectrometry, Sc plants were separate, and Vv plants and Vs hybrids had some degree of overlap, although they could still be distinguished.

As some compounds have isomers, the first-order mass spectra could not firmly identify all compounds, and each substance could be shattered in the mass spectrometer. These fragments could be detected by mass spectrometry to generate second-order mass spectra, which could be matched and scored with the second-order mass spectra of the standard substance in our in-house database. Finally, the second-order identification results of the metabolites were obtained. All metabolites, as well as comparative analysis and KEGG annotations of Vv-Vs, Sc-Vs and Vv-Sc, are listed in Tables S7–S9. The heatmap of different metabolites is shown in Figure 5B.

Through metabolomic analysis, we found that one term (Table S9, M553T311) for the enrichment of gomisin was up-regulated in Vs hybrids. This component is a medicinal ingredient in *S. chinensis* and has not been identified in any DEGs associated with these two substances; thus, the difference in this content may be caused by direct product transport. There were seven terms related to flavonoid metabolism with significant differences in Vv plants compared to Vs hybrids in the positive mass, and three in the negative mass, which were related to fruit color. As described above, there were also significant differences in fruit color in Vs hybrids and Vv plants. For all significantly different metabolites, the screening software MBRole (http://csbg.cnb.csic.es/mbrole/, accessed on 14 October 2020) was used for functional enrichment analysis, and the positive mass spectrometry results of Vv-Sc, Vv-Vs, and Sc-Vs are listed in Tables S7–S9. These results demonstrate that differences between Vv plants and Vs hybrids were minimal at approximately 30 items, with the three most frequent categories being purine metabolism, arginine and proline metabolism, and ATP-binding cassette transporters. The Sc-Vs and Sc-Vv datasets, respectively, had 68 and 65 different entries. Among the 30 significant categories in Vv-Vs, 27 had marked differences in Sc-Vv, and 25 had equal or greater KEGG-enriched compounds in Sc-Vv.

## 4. Discussion

Graft hybridization is a simple and efficient breeding method for woody plants, especially for reproductive isolation and highly heterozygous genotypes [4,39,40,41]. Here, we grafted two species with very distant relationships (*V. vinifera* and *S. chinensis*) and compared them to self-grafted grapes to demonstrate the changes in genetic material, plant phenotypes, and metabolite levels caused by the use of different rootstocks for the scion.

Through transcriptome sequencing and sRNA-seq, differences in gene expression and miRNA levels between Vs hybrids and Vv plants were determined. Our results demonstrate that Vs hybrids and Vv plants displayed significant differences in mRNA and miRNA levels associated with defense responses, oxidation-reduction processes, signal transduction, photosynthesis, and metabolism. The down−regulation of defense-related genes may play a key role in the survival of distant hybrid offspring without immune rejection. Furthermore, the down−regulation of auxin, gibberellin (GA), and other growth-related genes may lead to superior growth and development in Vv plants compared to the shorter internodes and altered morphology of leaves and fruits in Vs hybrids [42,43,44,45].

Fruit quality is the most critical factor determining the economic benefit of new varieties. As such, we analyzed genetic changes in the Vs hybrid that are related to fruit development, such as changes in fruit taste, size, shape, and color. Our transcriptome and small RNA sequencing results revealed that grafting of phylogenetically distant plants might induce defense reactions and signal transduction-related genes, which likely influences graft compatibility. Metabolomic results also showed that secondary metabolites from the rootstock could be transferred to the scion, as demonstrated by a higher content of gomisin and flavonoids in Vs compared to Vv.

### 4.1. Change in Defense-Related and Oxidation/Reduction Genes and miRNAs between Vv Plants and Vs Hybrids

The GO clustering analysis showed that the oxidation-reduction process accounts for the largest proportion of DEGs, and the analysis of DEMs showed that the proportion of DEGs related to defense responses was the largest. Both defense responses and oxidation-reduction processes play important roles in helping plants cope with stress and adverse physiological responses between the grafting partners. This is a common phenomenon and an important aspect of grafting incompatibility [46,47]. As such, the lower survival of Vs hybrids may be due to the down-regulation of defense-related genes and increased susceptibility to biotic and abiotic threats. A total of 31 DEGs were associated with defense response, 19 of which were down-regulated in Vs hybrids compared to Vv plants. Most of the defense-related genes were involved in signal transduction, and four genes with *p* < 0.005 were involved in intracellular signal transduction. Among the 131-oxidation-reduction process-related DEGs, 85 were down-regulated in Vs hybrids compared to Vv plants. GO analysis of miRNAs detected six DEMs associated with the defense response and two DEMs related to the oxidation-reduction process.

The *DXR* (*MSTRG.9167*) gene encodes 1-deoxy-d-xylulose-5-phosphate reductoisomerase, an important enzyme involved in the 2-C-methyl-d-erythritol-4-phosphate (MEP) pathway. Plants are sensitive to down−regulation of the *DXR* gene, and its mutation could lead to abnormal thylakoid whitening, dwarfed stature, and abnormal development of leaf trichomes [48]. Furthermore, *DXR* transcript levels regulate the expression levels of related genes; thus, they affect the biosynthesis of carotenoids, chlorophyll, GA, and abscisic acid [48]. Therefore, we believe changes in GA and auxin-related genes in Vs hybrids may have resulted in shorter internodes than in Vv plants. *DXR* transcription levels were significantly down−regulated in Vs hybrids, which may reduce the photosynthetic efficiency of Vs hybrids leaves and affect multiple hormone signaling pathways. *THI1-1* (*MSTRG.11573*) is a gene annotated as a thiamine thiazole synthase 1, chloroplast-like in grapes, and might play additional roles in adaptation to various stress conditions and DNA damage tolerance [49,50]. Thus, the down−regulation of this gene in Vs hybrids might be related to adaptation and defense responses caused by distant grafting. These data indicate a misregulation of defense and signaling pathways in plants grafted to phylogenetically distant partners.

### 4.2. Signal Transduction and Hormone-Related RNA Levels Are Significantly Different between Vs Hybrids and Vv Plants

Signal transduction pathways might play a vital role in graft establishment and account for a considerable proportion of DEMs and DEGs. *AUX1* is an auxin transporter protein gene found in field mustard, *Brassica rapa*, where its overexpression strongly promotes the growth and biomass production of seedlings and plants [51]. *AUX1* was significantly down−regulated in Vs hybrids (the FPKM in Vv plants was 39.67 and 19.09 in Vs hybrids).

*GA3* (*MSTRG.10120*) was significantly down-regulated in Vs hybrids; the FPKM in Vv plants was approximately 12, and approximately 0 in Vv hybrids. *GA3* encodes ent-kaurene oxidase, which is the first cytochrome P450-mediated step in the GA biosynthetic pathway [52,53]. Its GO term contains the GA-mediated signaling pathway, as well as the GA biosynthetic process. Thus, the down-regulation of this gene in Vs hybrids may affect the synthesis of GA and the accumulation of superoxide. *GA3*, which encodes the first cytochrome P450-mediated step in the GA biosynthetic pathway [53,54], was down-regulated in Vs hybrids (see Section 4.1). *GAI1* (*MSTRG.412*) is a gene regulated by ethylene that negatively regulates the GA signaling pathway [55] and is significantly up-regulated over 18-fold in Vs. The down−regulation of *GA3* and up−regulation of *GAI1* suggest that the GA signaling pathway was likely blocked in Vs hybrids. Both auxin and GA are hormones that promote plant growth and development [56,57]. Their down−regulation in Vs hybrids might be detrimental to plant growth. The *BSK1* gene participates in BR signaling, regulates the link between plant growth and defense [58], and is significantly up−regulated in Vs hybrids. Significant differences in the expression levels of these genes suggest that there are likely genetic changes in growth, signaling, and defense responses in Vs hybrids. Auxin and jasmonic acid promote *BSK1* expression, and abscisic acid inhibits *BSK1* expression [59]. *BSK1* was significantly up-regulated in Vs hybrids (8.11-fold), which may indicate changes in related hormone levels in Vs hybrids. Taken together, our transcriptomic data suggest that hormone homeostasis is severely affected by grafting phylogenetically-distant plants.

### 4.3. DEGs and DEMs Associated with Photosynthesis and Fruit Development

Leaf color, leaf shape, and stem color of *S. chinensis* in Vs hybrids exhibited obvious changes (Figure 1). This is likely the result of the combined effects of diverse genetic backgrounds, differential hormonal regulation, physiological conditions, and environment. The main function of the leaf is photosynthesis, so we focused on photosynthesis-related gene expression level changes. Sixty photosynthesis-related transcripts displayed significant differences between Vv plants and Vs hybrids, with 45 down-regulated transcripts in Vs hybrids. Among them, 12 DEGs were associated with pigment binding, chloroplasts, and light harvesting. Nine DEGs were down−regulated in Vs hybrids and *MSTRG.11440*, *MSTRG.21184*, and *MSTRG.9394* were up−regulated.

Although most of the light harvesting-related genes were down-regulated in Vs hybrids, their expression levels were relatively low (the average FPKM in Vv plants was typically less than 10, with only two greater than 30 and below 50), but the up−regulated gene *MSTRG.9394* had an FPKM ten-fold higher than the FPKM for *MSTRG.21184* in Vs. These two up-regulated genes may play an important role in the photosynthesis process of Vs hybrids. The genes *LHCA5* (*MSTRG.10071*), *LHCA2* (*MSTRG.1081*), *LHCA1* (*MSTRG.4497*), *LHCA4* (*MSTRG.8983*)*,* and *LHCB5* (*MSTRG.10998*), which participate in the light-harvesting complex of photosystem I and photosystem II [60], were all down-regulated in Vs hybrids. This suggests that the light-capturing ability of Vs hybrids might be lower than that of Vv plants.

The fruit size, taste, and color of Vs were significantly different from that of Vv. Compared to Vv plants, the fruits of Vs hybrids were significantly smaller and had a deeper red coloration. Of the six DEGs participating in the flavonoid biosynthetic process, *MSTRG.6465* and *MSTRG.2147* were up−regulated in Vs hybrids compared to Vv plants, while the other four DEGs were down-regulated. Target gene prediction and GO annotation revealed miRNAs (*vvi-miR167c-p3*) connected to the flavonoid biosynthetic process (*vvi-MIR159c-p5*) and related to fruit development and leaf development. Auxin and GA both regulate fruit development [10,61]. As such, the differential genetic material of the auxin and GA signaling pathway we mentioned above may also influence the phenotype of the fruit.

### 4.4. Metabolism-Related DEGs and DEMs and Comprehensive Analysis of Distant Grating-Related Communication of Biomolecules

KEGG analysis detected 147 metabolism-related DEGs, mainly associated with carbon metabolism, nitrogen metabolism, lipid metabolism, purine metabolism, pyrimidine metabolism, thiamine metabolism, and sulfur metabolism. Our metabolomic analysis showed that pyrimidine metabolism, porphyrin and chlorophyll metabolism, and cysteine and methionine metabolism were the most diverse compounds in Vs hybrids and Vv plants, and they had seven, nine, and eight DEGs, respectively. This suggests that changes in metabolites due to grafting hybridization might be caused at the transcriptional level.

This study demonstrates that phylogenetically distant grafting caused significant changes in the transcriptome and metabolites. However, the differences caused by the transport between phloem-mediated transport of mRNA [6] and were caused by changes in scion transcriptome remain unclear. Furthermore, their differentiation patterns during plant growth and development during grafting require further study. The correlation of major differences in metabolites and related genes indicates that the differences in metabolomes are controlled at the transcriptional level to an extent. Some differences in metabolites of *S. chinensis* indicate that metabolites might be transported directly from the rootstock to the scion after adhesion, and this transport of metabolites between rootstocks and scions and its significance to plant growth deserves further study.

## 5. Conclusions

The variation in phenotypic traits, including those of leaves, internodes, and fruits in vegetative hybrids, tend to be at an intermediate state between those of the rootstock and scion, with a tendency toward rootstock-like traits in *Vitis vinifera*/*Schisandra chinensis* (Figure 1). We observed differences between genes, miRNAs, and metabolites in *Vitis vinifera*/*Schisandra chinensis* and self-rooted *V. vinifera*. In our study, there were 2112 DEG with *p* values < 0.05 and 222 DEG with *p* values ≤ 0.005. Furthermore, the levels of 79 miRNAs were significantly different in Vv plants and Vs hybrids, which included 27 known miRNAs and 52 new ones. DEGs and DEMs functioned mainly focused on oxidation-reduction processes, defense responses, metabolic processes, signal transduction, ATP binding, and similar functions. Through metabolomic analysis, we found that one-term enrichment of Schisandra was significantly down-regulated, and that of gomisin was up-regulated in Vs hybrids. These two components are the main medicinal ingredients in *S. chinensis* and have not been found in any DEGs associated with these two substances. Thus, the difference in their content might be caused by direct product transportation between stock and scion. The above results indicate that the rootstock genotype had a substantial influence on the transcriptome and metabolome of the scion, and the hybrid traits might be able to be tailored based on the selection of target rootstocks.

## Figures and Tables

**Table 1:** Summary of Illumina transcriptome sequencing data for Vv and Vs.

Type	Vv	Vs	Sum
Raw Reads	138,096,216	153,048,950	291,145,166
Valid Reads	136,975,222	151,704,222	288,679,444
Valid Reads Ratio (%)	99.19	99.11	/
Mapped Reads	103,135,533	100,706,990	203,842,532
Q20 (%)	98.98	99.12	/
GC Content (%)	48.67	50.17	/
Member of Transcript	/	/	30,609
GO Annotate	/	/	21,109
KEGG Annotate	/	/	4151

**Table 2:** Summary of metabolome results.

Mode	Comparison	All	Up	Down
Pos	Vv/Vs	11,757	734	454
Pos	Sc/Vv	11,757	2232	2099
Pos	Sc/Vs	11,757	2299	1911
Neg	Vv/Vs	7868	353	260
Neg	Sc/Vv	7868	1423	1360
Neg	Sc/Vs	7868	1522	1255

## Data Availability

The RNA sequencing raw data were deposited in the Gene Expression Omnibus at the National Center for Biotechnology Information website (GEO, https://www.ncbi.nlm.nih.gov/geo accessed on 10 August 2023) with the accession number GSE130209.

## Supplementary Materials

The following supporting information can be downloaded at https://www.mdpi.com/article/10.3390/plants13121676/s1. Table S1: Sequence of transcripts or miRNAs and primers performed to verify the sequencing results which have been used for RT-qPCR; Table S2: Results of differentially expressed genes identified by sequencing, including gene ID, expression level and function; Table S3: Results of differential expression of miRNAs; Table S4: GO Enrichment of the differential miRNA target gene by psRNATarget prediction; Table S5: The sequencing results of the target genes predicted by miRNA that were detected by degradation; Table S6: The statistic of the target genes predicted by miRNA that were detected by degradation; Table S7: Metabolitome results of Vv/Vs in anion mode; Table S8: Metabolitome results of Sc/Vs in cationic mode; Table S9: Metabolitome results of Sc/Vv in cationic mode.
